# Can polysaccharide K improve therapeutic efficacy and safety in gastrointestinal cancer? a systematic review and network meta-analysis

**DOI:** 10.18632/oncotarget.19059

**Published:** 2017-07-06

**Authors:** Yan Ma, Xiaofen Wu, Jingwen Yu, Jinyan Zhu, Xia Pen, Xianjun Meng

**Affiliations:** ^1^ College of Food Science, Shenyang Agriculture University, Shenyang 110866, P.R. China; ^2^ Center of Experiment Teaching, Shenyang Normal University, Shenyang 110034, P.R. China; ^3^ The Sixth People’s Hospital of Shenyang, Shenyang 110005, P.R. China; ^4^ Food Inspection Monitoring Center of Zhuanghe, Dalian 116400, P.R. China

**Keywords:** polysaccharide K, PSK, immunochemotherapy, gastrointestinal cancer, network meta-analysis

## Abstract

**Objective:**

To assess the comparative efficacy and safety of polysaccharide K (PSK), with or without chemotherapy, for patients with gastrointestinal cancer (GIC) through a systematic review and network meta-analysis.

**Materials and Methods:**

We performed a network meta-analysis to identify evidence from randomized controlled trials. We searched PubMed, Embase and the Cochrane Library for publications up to May 2017. The prespecified primary efficacy outcomes were 1–7 year overall survival (OS), while the secondary efficacy outcomes were 1–7 year disease-free survival (DFS); we performed subgroup analyses and meta-regressions according to the cancer type (colorectal, esophagus and gastric cancer) and treatment arms (with or without chemotherapy). Safety outcomes were side effects of PSK. We conducted pairwise meta-analyses using a random-effects model and then performed random-effects network meta-analyses.

**Results:**

A total of 23 trials were eligible, involving 10684 patients and 13 intervention arms. PSK treatment significantly increased 1–5 year OS and resulted in positive trends in 6–7 year OS; significant increases were also found in 1–7 year DFS, while no increase in side effects was observed. Significant efficacy outcomes obvious in colorectal and gastric cancer groups, as well as PSK combined with chemotherapy groups (iv, po, iv+po). Network meta-analysis revealed that PSK combined with chemotherapy was superior, with significantly increased 3-year and 5-year OS. The study is registered with PROSPERO (CRD42017065193)

**Conclusions:**

The adjuvant immunochemotherapy agent PSK is effective and safe for patients with GIC. PSK combined with chemotherapy appears to be the preferred application of PSK.

## INTRODUCTION

Gastrointestinal cancers (GICs) include several types of cancer, including liver, colorectal, pancreatic, gastric and esophageal cancer, and present an increasing global public health threat. GICs account for approximately 30% of all cancers worldwide. Most are characterized by a remarkable male predominance in incidence [1–2]. Colorectal cancer (CRC) is the second leading cause of cancer-related death in the United States and the third most common malignant cancer worldwide; gastric cancer and esophageal cancer are relatively rare but have poorer prognoses, with only 30.4% and 18.4% 5-year survival rates in the United States, respectively [3–4]. Because primary GIC exhibits an insidious onset, rapid development and a high degree of malignancy, early clinical diagnosis is difficult. Many patients who are symptomatic at presentation already have late cancer, with local and distant metastasis; radiotherapy and chemotherapy are not sensitive and postoperative relapse is common, which are the major reasons for the high mortality rate [5–6]. Although recent developments in treatment modalities, including surgical resection, have significantly improved the long-term survival of patients with GIC, the overall prognosis remains poor. Therefore, new and adjuvant treatments are needed in clinical practice.

Polysaccharide K (PSK) is a protein-bound polysaccharide that is extracted from the mycelia of *Coriolus versicolor* strain CM-101, one of the basidiomycetes [7]. It has a mean molecular weight of 9.4 × 10^4^ and is composed of a glucan with a β1–β4 bond in the main chain and β1–β3 and β1–β6 bonds in the side chain; the latter binds to a protein moiety through O- or N-glycoside bonds [8]. The constituent major monosaccharide is glucose, with smaller amounts of other saccharides (Supplementary Table 1A); the protein portion of PSK consists predominantly of acidic amino acids such as aspartic acid and glutamic acid, along with neutral amino acids such as valine and leucine and basic amino acids including small amounts of lysine and arginine (Supplementary Table 1B). The possibility of biological modulation was raised by the observation that PSK acts on immunocytes and potentiates the activity of interferon, interleukin-2, and NK cells [9]; PSK is a potent inducer of gene expression for some interleukins (interleukin -1a, -p, -6, -8) as well as tumor necrosis factor-a and monocyte chemotactic and activating factors [10]. These observations remain controversial and require additional research.

Beneficial therapeutic effects of PSK with or without chemotherapy have been demonstrated in a series of clinical studies of patients with GIC, while others did not yield significant results. A pairwise meta-analysis [11] assessed the impact of PSK on survival rates but only considered direct evidence and did not investigate which PSK treatment arm was most appropriate. No previous reviews have provided a comprehensive overview including network meta-analysis and meta-regression.

## RESULTS

### Systematic review and qualitative assessment

**Figure 1:**
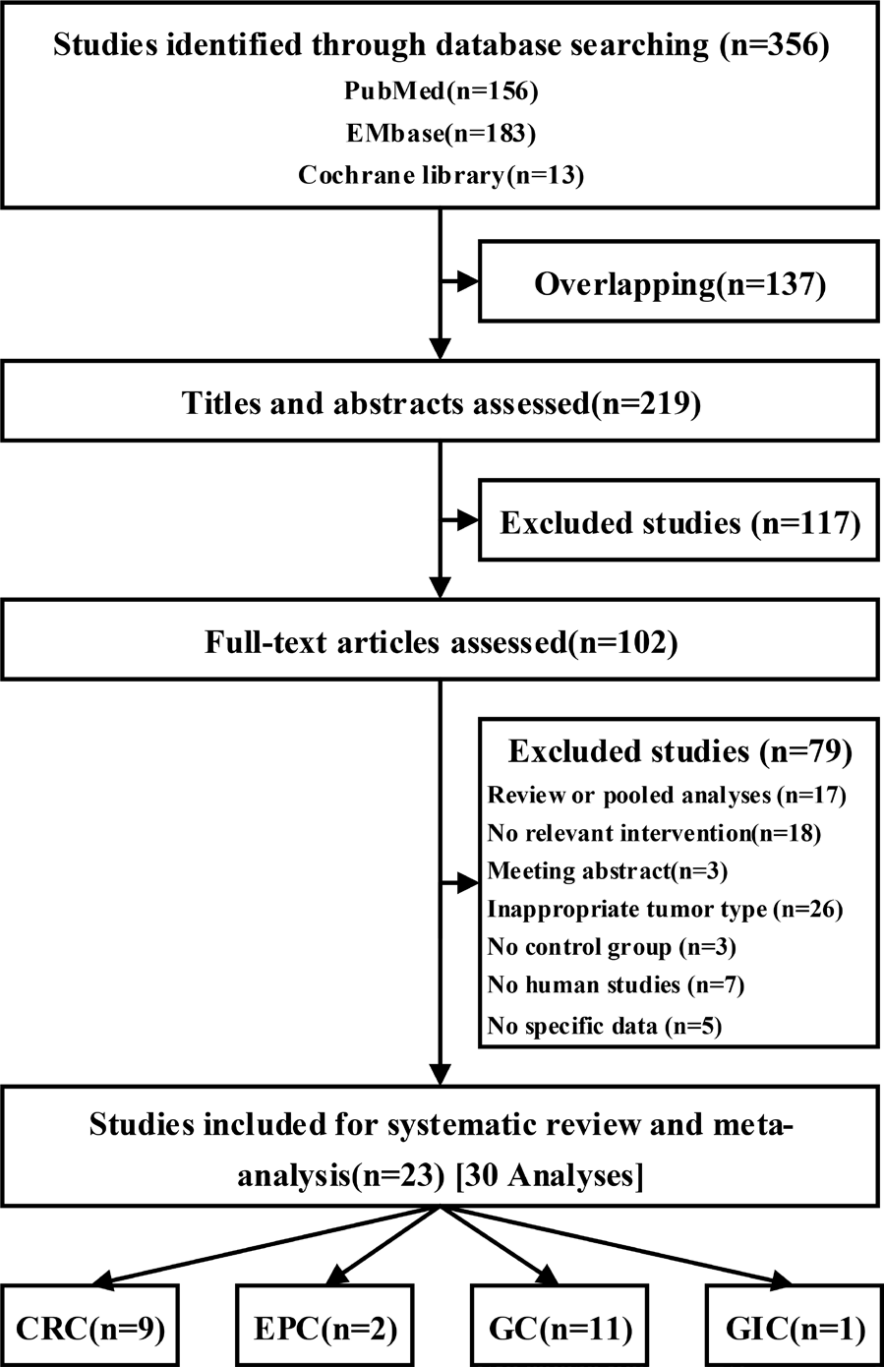
Flow of studies through review process for systematic review and meta-analysis

A total of 219 unique citations were identified using our search strategy. Of these, 23 [12–34] trials (30 analyses; *n* = 10684) and 13 interventions [PSK plus chemotherapy (CT) vs chemotherapy alone (po) (PSK plus 5-Fu vs 5-Fu alone, PSK plus UFT vs UFT alone, PSK plus S-1 vs S-1 alone, PSK plus Tegafur vs Tegafur alone, and PSK plus Futraful vs Futraful alone); PSK plus chemotherapy vs chemotherapy alone (po+iv); PSK plus chemotherapy and immunochemotherapy (ICT) vs chemotherapy and immunochemotherapy (po); PSK plus radiation (RT) vs radiation; PSK plus radiation and immunochemotherapy vs chemotherapy and radiation; PSK plus chemotherapy (iv) vs PSK plus chemotherapy (po); PSK vs chemotherapy alone (po) and PSK vs placebo] were included (Figure 1). The mean trial sample size was 464, ranging between 21 and 2764 patients. Dates of publication ranged from 1988 to 2013. Overall, 5543 patients were randomly assigned to the PSK group and 5141 to the control group. Nine trials included patients with CRC, 2 with EPC, 11 with GC and 1 with GIC.

Table 1 summarizes the differences in the fundamental characteristics between the PSK arm and the control arm (see the full list of characteristics in Supplementary Table 2). These statistics revealed that the two arms were similar in age, gender, tumor stage and histological grade. In the baseline characteristics analysis, we did not group trials by cancer type or treatment arm; the baseline characteristics were balanced between the PSK arm and the control arm. Quality assessments of this study are presented in Supplementary Table 3; the PEDro score indicated that all included trials were of acceptable quality.

**Table 1:** Characteristics of baseline in patients associated with the PSK arm vs the control arm

	PSK arm vs control arm (OR, 95%CI)	Heterogeneity
**Age (year)**	−0.00 (−0.14, 0.13)*	*P* = 0.010, *I*^2^ = 52.2%
**Male**	0.93 (0.82, 1.05)	*P* = 0.797, *I*^2^ = 0.0%
**Tumor stage (I–II/III–IV)**	1.07 (0.90, 1.28)	*P* = 0.861, *I*^2^ = 0.0%
**Histological grade (well/moderate+poor)**	0.90 (0.71∼1.16)	*P* = 0.108, *I*^2^ = 31.8%

*Standardized mean difference; PSK, Polysaccharide K.

**Figure 2:**
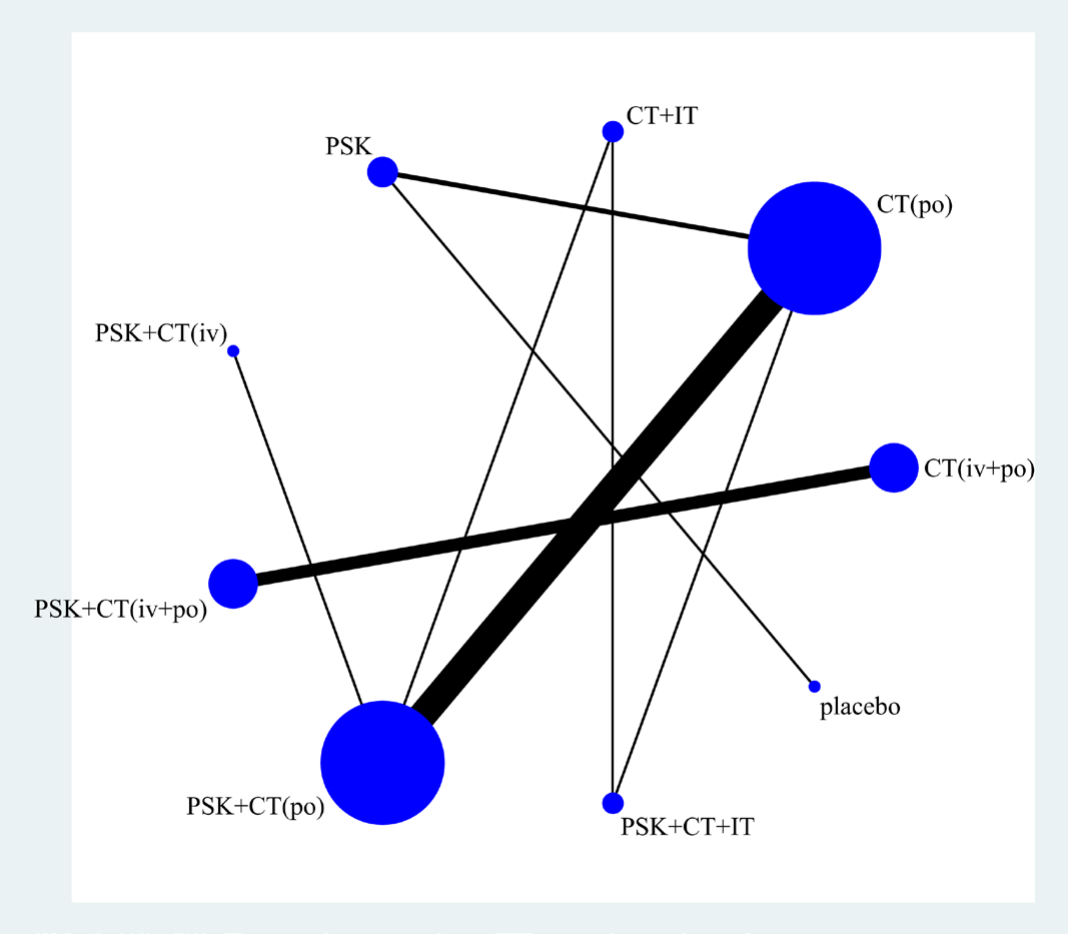
Network of eligible comparisons for 3-year overall survival The width of the lines is proportional to the number of trials comparing every pair of treatments, and the size of every circle is proportional to the number of randomly assigned participants (the sample size). Abbreviation: CT, chemotherapy; IT, immunochemotherapy; PSK, Polysaccharide K.

Figure 2 displays the network weight of eligible comparisons for 3-year overall survival displaying the available direct comparisons and network of the trials. Table 2 summarizes the numbers of patients with GIC according to study treatment. Patients were grouped by different treatment arms (most trials only had two arms). More than half of our included trials [12–17, 20, 24–25, 27–30, 32] compared the efficacy and safety of PSK plus chemotherapy with chemotherapy alone (po).

**Table 2:** Number of patients with gastrointestinal cancer according to study treatment

Treatment arms	Number of trials	Participants (% Total)		Cancer type
		Group 1	Group 2	
**PSK plus chemotherapy vs chemotherapy alone (po)**	14 [12–17, 20, 24–25, 27–30, 32]	2874 (51.85%)	2601 (50.59%)	7CRC, 7GC
PSK plus 5-Fu vs 5-Fu alone	4 [12–13, 20, 29, 33]	686 (12.38%)	668 (12.99%)	3CRC, 2GC
PSK plus UFT vs UFT alone	4 [14–16, 24]	418 (7.54%)	214 (4.16%)	3CRC, 1GC
PSK plus S-1 vs S-1 alone	2 [17, 27]	25 (0.45%)	25 (0.49%)	1CRC, 1GC
PSK plus Tegafur vs Tegafur alone	2 [25, 28]	1554 (28.04%)	1475 (28.69%)	2GC
PSK plus Futraful vs Futraful alone	2 [30]	191 (3.45)	199 (3.87%)	GC
**PSK plus chemotherapy vs chemotherapy alone (po+iv)**	3 [18, 26, 31, 33]	593 (10.70%)	601 (11.69%)	1CRC, 3GC
**PSK plus chemotherapy and immunochemotherapy vs chemotherapy and immunochemotherapy (po)**	1 [25]	1357 (24.48%)	1363 (26.51%)	GC
**PSK plus radiation vs radiation**	2 [21–22]	105 (1.89%)	90 (1.75%)	2EPC
**PSK plus radiation and immunochemotherapy vs chemotherapy and radiation**	2 [21–22]	74 (1.34%)	63 (1.23%)	2EPC
**PSK plus chemotherapy (iv) vs PSK plus chemotherapy (po)**	1 [23]	44 (0.79%)	38 (0.74%)	1GC
**PSK vs chemotherapy alone (po)**	2 [28, 30]	307 (5.54%)	316 (6.15%)	2GC
**PSK vs placebo**	2 [19, 34]	89 (1.61%)	69 (1.34%)	1CRC, 1GIC
**Total**	23 [12–34]	5543	5141	9CRC, 2EPC, 11GC, 1GIC

5-Fu, 5-fluorouracil; PSK, Polysaccharide K; UFT, Tegafur/uracil.

### Pairwise meta-analysis–efficacy outcomes

Supplementary Table 5 summarizes the results of 1-year to 7-year overall survival (OS) and disease-free survival (DFS) associated with the PSK arm vs the no PSK control arm.

### Overall survival

The PSK arm was associated with a significant increase in 1-year OS (OR: 1.59, 95% CI: 1.12 to 2.25) accompanied by modest heterogeneity (*P* = 0.012, *I*^2^ = 45.8%). We calculated the *P* value by meta-regression, and the origin of heterogeneity may vary according to cancer type (*P* = 0.000) or treatment arm (*P* = 0.006). A similar effect value could be found in subgroups of CRC, EPC, PSK+CT vs CT alone (po) and PSK+RT+CT vs RT+CT. Sub-treatment of PSK+CT vs CT alone (po)–PSK plus UFT vs UFT alone group also revealed similar results. Furthermore, the PSK arm was associated with a significant increase in 2-year OS (1.43, 1.17 to 1.75) accompanied by modest heterogeneity (*P* = 0.083, *I*^2^ = 31.0%). Different cancer types (*P* = 0.000) and treatment arms (*P* = 0.004) may still be sources of heterogeneity by meta-regression. Similar effect values were observed in subgroups of CRC, GC, PSK+CT vs CT alone (po) and PSK+CT vs CT alone (iv+po). Sub-treatment of PSK+CT vs CT alone (po)–PSK plus 5-Fu vs 5-Fu alone and PSK plus UFT vs UFT alone groups also revealed similar results. Moreover, for 3-year OS, significant results were observed overall (1.35, 1.14 to 1.59) and for CRC, GC, PSK+CT vs CT alone (po) and PSK+CT vs CT alone (iv+po). Sub-treatment of PSK+CT vs CT alone (po)–PSK plus 5-Fu vs 5-Fu alone and PSK plus UFT vs UFT alone subgroups. The *P* values from the meta-regression revealed that different cancer types (*P* = 0.000) and treatment arms (*P* = 0.014) had greater impact on the efficacy results.

In the 4-year follow up, OS could be significant enhanced overall (1.41, 1.15 to 1.73) in the subgroup of CRC, GC, PSK+CT vs CT alone (po) and PSK+CT vs CT alone (iv+po). Sub-treatment of PSK+CT vs CT alone (po)–PSK plus UFT vs UFT alone. The *P* value of the meta-regression revealed that different cancer types (*P* = 0.016) and treatment arms (*P* = 0.003) have greater impact on the efficacy. Furthermore, significant improvement in 5-year OS in the PSK arm of total (1.37, 1.22 to 1.68) and subgroups of GC, PSK+CT vs CT alone (po) and PSK+CT vs CT alone (iv+po) was observed; similar observations were evident in the sub-treatment of PSK+CT vs CT alone (po)–PSK plus 5-Fu vs 5-Fu alone and PSK plus UFT vs UFT alone. Meta-regression revealed that substantial heterogeneity (*P* = 0.001, *I*^2^ = 55.3%) did not stem from the differences in cancer types and treatment arms. Moreover, in the sixth year, no significant results favoring the PSK arm could be found (1.14, 0.73 to 1.79) with substantial heterogeneity (*P* = 0.002, *I*^2^ = 71.3%). Meta-regression did not find the source of heterogeneity; however, in the subgroup of PSK+CT vs CT alone (iv+po), positive results were observed. The least compelling result was for 7-year OS, in which favorable results for PSK appeared in subgroups–PSK plus 5-Fu vs 5-Fu alone and PSK plus UFT vs UFT alone. The total result did not show significance (1.35, 0.93 to 1.94), with low heterogeneity (*P* = 0.027, *I*^2^ = 57.9%).

In general, our pairwise meta-analysis suggested that OS was significantly improved from 1-year to 5-year survival, especially in CRC, GC, PSK+CT vs CT alone (po) and PSK+CT vs CT alone (iv+po), as well as sub-treatment of PSK+CT vs CT alone (po)–PSK plus 5-Fu vs 5-Fu alone and PSK plus UFT vs UFT alone subgroups. Little evidence of bias could be found in Begg's and Egger's tests, with moderate to high quality evidence according to the GRADE assessment. However, this conclusion was not well supported; meta-regression results also showed that the difference between the two arms was variable (*P* < 0.05), so we need to perform further comparisons by network meta-analysis.

### Disease-free survival

The PSK arm was associated with a significant increase in 1-year DFS (1.37, 1.04 to 1.81) accompanied by low heterogeneity (*P* = 0.488, *I*^2^ = 0.0%). A similar effect was found in subgroups of CRC and PSK plus UFT vs UFT alone. Furthermore, significant differences were observed in total (1.68, 1.36 to 2.07) and CRC, PSK plus 5-Fu vs 5-Fu alone and PSK plus UFT vs UFT alone subgroups. Moreover, significant differences were seen in 3-year DFS for total (1.58, 1.30 to 1.92) and all subgroups; 4-year DFS at total (1.73, 1.43 to 2.10) and all subgroups; 5-year DFS at total (1.74, 1.43 to 2.11) and all subgroups; which all favor the PSK arm. The smallest differences were seen in 6-year DFS (1.49, 1.11 to 2.00) and 7-year DFS (1.66, 1.11 to 2.48), both favoring the PSK arm overall.

In general, our pairwise meta-analysis suggested that DFS significantly improved from 1-year to 7-year survival, both in total and subgroups. Meta-regression showed no significant difference according to the two group modes. Little evidence of bias could be found in Begg’s and Egger’s tests, with moderate to high quality evidence according to the GRADE assessment.

### Network meta-analysis–efficacy outcomes

**Figure 3:**
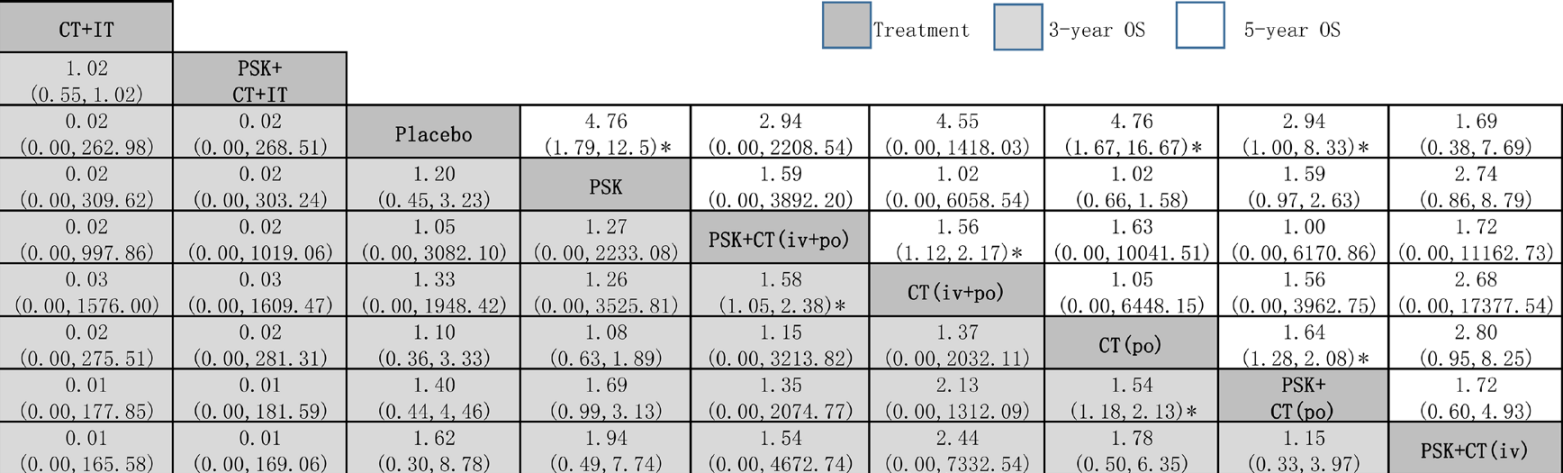
Network meta-analysis of 3-year OS and 5-year OS The treatments are reported in order of survival rate ranking according to SUCRA of 3-year OS. Comparisons should be read from left to right. The estimate is located at the intersection of the column-defining the treatment and the row-defining the treatment. For OS, a OR value above 1 favors the PSK arm. *Result with significant difference. Abbreviation: CT, chemotherapy; IT, immunochemotherapy; OS, overall survival; PSK, Polysaccharide K.

Because different cancer types and treatment arms had greater impacts on the pairwise meta-analysis results (*P* < 0.05 by meta-regressions), we wanted to find the most effective treatment arm. We therefore compared more representative and controversial data through network meta-analysis: 3-year and 5-year OS (excluding EPC). The results of the network meta-analysis for 3-year and 5-year OS are presented in a two-league table (Figure 3).

### 3-year OS

Network meta-analysis suggested that, compared with placebo, PSK+CT (iv) was ranked best for improving 3-year OS (1.62, 0.30 to 8.78), followed by PSK+CT (po) (1.40, 0.44 to 4.46), CT (po) (1.10, 0.36 to 3.33, CT (iv+po) (1.33, 0.00 to 1948.42), PSK+CT (iv+po) (1.05, 0.00 to 3082.10) and PSK (1.20, 0.45 to 3.23), all of which ranked higher than usual placebo. The only two exceptions were PSK+CT+IT and CT+IT, which ranked lower than placebo. However, the evidence was based on only one trial of relatively poor quality that was not recently published [25], which is a major limitation to the reliability of our findings.

When we assessed the comparative efficacy, PSK+CT (iv) was superior to all other treatment arms; however, all of the treatment arms did not reach significance. Treatment arms were comparable in terms of improvement in 3-year OS, with significant differences in PSK+CT (po) vs CT (po) alone group (1.54, 1.18 to 2.13) and PSK+CT (iv+po) vs CT (iv+po) alone group (1.58, 1.05 to 2.38).

### 5-year OS

Network meta-analysis suggested that, compared with placebo, PSK+CT (po) was significantly superior in 5-year OS (2.94, 1.00 to 8.33), followed by CT (po) (4.76, 1.67 to 16.67), PSK (4.76, 1.79 to 12.5), PSK+CT (iv+po) (2.94, 0.00 to 2208.54), CT (iv+po) (4.55, 0.00 to 1418.03) and PSK+CT (iv) (1.69, 0.38 to 7.69).

When we assessed the comparative efficacy, PSK+CT (po) was superior to all other treatment arms including PSK. However, except for the CT (po) arm and PSK arm, the other treatment arms did not reach significance. Treatment arms were comparable with each other for improvement of 5-year OS, with significant difference found in PSK+CT (iv+po) vs CT (iv+po) alone group (1.56, 1.12 to 2.17).

### Pairwise meta-analysis–safety outcomes

Table 3 summarizes results of the toxicity associated with the PSK arm vs the control arm. We found that PSK not only did not increase the risk of side effects but also reduced the occurrence of nausea and vomiting (0.53, 0.31 to 0.91) and leukopenia (0.60, 0.43 to 0.83). Moreover, the use of PSK was safe, with mostly moderate to high quality evidence according to GRADE assessment.

**Table 3:** Meta-analysis for the toxicity associated with PSK arm vs control arm

Toxicity	Patients (P/C)	OR (95%CI)	Heterogeneity (*P*, *I*^2^)	Quality of evidence
***Gastrointestinal***				
Nausea-vomiting	6 (685/701)	0.53 (0.31, 0.91)*	*P* = 0.195, *I*^2^ = 32.1%	High
Diarrhea	6 (685/701)	0.84 (0.50, 1.40)	*P* = 0.264, *I*^2^ = 22.6%	High
Anorexia	5 (670/686)	0.89 (0.55, 1.45)	*P* = 0.259, *I*^2^ = 24.4%	High
Obstipation	2 (276/276)	1.42 (0.85, 2.37)	*P* = 0.331, *I*^2^ = 0.0%	Moderate
***Haematological***				
Anemia	3 (456/463)	0.86 (0.56, 1.30)	*P* = 0.389, *I*^2^ = 0.0%	High
Leukopenia	5 (629/646)	0.60 (0.43, 0.83) *	*P* = 0.3603, *I*^2^ = 8.1%	High
Thrombocytopenia	3 (359/365)	1.45 (0.94, 2.22)	*P* = 0.633, *I*^2^ = 0.0%	High
***Abnormal hepatic function***	5 (782/815)	0.93 (0.71, 1.20)	*P* = 0.524, *I*^2^ = 0.0%	High
***Abnormal pain***	3 (393/404)	0.69 (0.27, 1.79)	*P* = 0.053, *I*^2^ = 65.9%^#^	Moderate
***Stomatitis***	2 (269/275)	0.89 (0.03, 23.77)	*P* = 0.077, *I*^2^ = 68.0%^#^	Low

C, control group; P, PSK group; PSK, Polysaccharide.

*Result with significant differences; ^#^Substantial heterogeneity.

## DISCUSSION

This network meta-analysis represents the most comprehensive synthesis of data for currently available adjuvant immunochemotherapy with Polysaccharide K studies on patients with gastrointestinal cancer. We combined direct and indirect evidence from 23 randomized controlled trials (30 analyses) comparing 13 different interventions on 10684 patients with GIC to make several key observations regarding the potential efficacy and safety of GIC adjuvant immunochemotherapy agent. First, PSK arms were superior to non-PSK arms in overall survival, especially for colorectal cancer and gastric cancer groups. Furthermore, the treatment arms with PSK+chemotherapy showed significant advantages over one to seven years, with moderate to high confidence in estimates. Due to the large differences in treatment arms (by meta-regression), according to network meta-analysis of 3-year OS, PSK+chemotherapy (iv) had superior efficacy in terms of treatment and PSK+chemotherapy (po) had superior efficacy in 5-year OS. PSK arms were also superior to non-PSK arms in disease-free survival, and there was no significant difference between the different cancer types and treatment arms. Finally, PSK did not increase the risk of toxicity. Overall, compared with non-PSK arms, adjuvant immunochemotherapy with PSK was found to be both safe and efficacious.

Although the mechanisms of action of the effects of PSK administration have not been elucidated, two possible mechanisms are speculated. The first possibility is that the decrease in NK cells is milder with PSK administration because PSK alleviates leucopenia induced by chemotherapy in mice [35]. The second possibility is that PSK administration promotes the proliferation of NK cells, compensating for the decrease in the number of NK cells, based on the findings that PSK showed *in vitro* mitogenic action against peripheral blood mononuclear cells of healthy individuals [36], PSK promoted the proliferation of mouse splenic T-cells [37] and human NK cell line [38], and PSK promoted interleukin (IL)-2 production and increased IL-2 receptor expression in normal human lymphocytes [39].

Recently, a review by Maehara and colleagues evaluated OS in the treatment of colorectal cancer [11]. Our meta-analysis differs from the earlier study in several ways. First, the main objective of our study was to evaluate all existing treatment arms for adjuvant immunochemotherapy by PSK for GIC from randomized controlled trials, whereas Maehara and colleagues’ study evaluated only colorectal cancer. Second, the previous review did not conduct subgroup analyses and meta-regressions, which could not explain the substantial heterogeneity. Finally, the previous review only performed standard pairwise comparisons between the PSK arm and control arm. Our study extends the findings from network meta-analysis and ranked the treatment arms to determine the best option to consider when PSK is used.

This review followed the guidelines for conducting rigorous systematic reviews and network meta-analyses [41–43]. To identify as many relevant reports as possible and to decrease the risk of bias, a comprehensive search strategy was designed. Based on these considerations, we observed little evidence of publication bias by statistical assessment. The treatment by PSK significantly increased 1–5 years of OS and showed positive trends in 6–7 years OS and significantly increased 1–7 years of DFS; no side effects were increased. Subgroup analyses and network meta-analysis revealed that PSK combined with chemotherapy ranked best with significantly increased survival.

It is not difficult to understand that the PSK group has a significant effect in 1–5 year OS, and there was no significant effect in 6–7 year OS. This may be because the treatment efficiency of the long-term tumor survival drug cannot keep pace with tumor recurrence and the rate of deterioration. Subgroup analyses and network meta-analysis revealed that PSK combined with chemotherapy ranked best with significantly increased survival.

PSK is an adjuvant immunochemotherapy agent; although it has a significant therapeutic effect in patients with GIC, it may be more suitable for certain types of patients. Studies by Saji S [31] and Sakamoto J [32] declared that the lower the serum immunosuppressive acidic protein level (< 580/μg/ ml), the higher the survival rate. PSK would be most effective in patients whose preoperative serum immunosuppressive acidic protein level is lower than the threshold level. Toge T [33] found that PSK extended survival in the group of patients with a preoperative granulocyte and lymphocyte count ratio of > 2.0, perhaps through restoration of immunocompetence. Therefore, the therapeutic effect of PSK and the patient's immune function are related, although this relationship is unclear.

This network meta-analysis had some limitations that merit further discussion. First, the time range of the included publications was too broad, with the therapeutic effect reported in publications more than 20 years old possibly being less accurate. Furthermore, in some cases, the original language of the publications could not be obtained, which reduced the number of trials we included, and may have impacted the accuracy of the results. Furthermore, positive results are easy to publish, but negative results are unlikely to leave the laboratory. An additional limitation of pairwise outcomes is their extensive heterogeneity (Supplementary Table 5 and Table 3), which indicated substantial variability in the outcomes of the included studies, although this was often because of the presence of heterogeneity in the baseline outcomes (Table 1) and the differences observed in the cancer types and treatment arms. Finally, subject differences existed, as some patients were involved in post-operative trials while others did not undergo surgery, which may have had an impact on the results.

PSK is safe and efficacious adjuvant immuno-chemotherapy agent. Additional RCTs of PSK should include larger samples and be robust and randomized to confirm the effects and toxicity of PSK on patient-relevant or disease-specific outcomes, particularly in patients with GIC. Future studies should ensure that appropriate methods are used for randomization, blinding and intent-to-treat. Furthermore, trials should assess outcomes using standardized or prescribed measures at similar time points. Analyses of individual data will be valuable for further exploration. More normative studies should be utilized in future network meta-analyses.

The finding of this comprehensive network meta-analysis provides some evidence that PSK might improve overall survival and disease-free survival without increasing side effects. On a local scale, patients with gastrointestinal cancers could be encouraged to accept PSK for adjuvant immunochemotherapy, especially combined with chemotherapy. In the clinical therapy of patients with GIC, PSK can be used as a first-line adjuvant immunochemotherapy agent.

## MATERIALS AND METHODS

### Search strategy and selection criteria

This systematic review was performed with an a priori established protocol (PROSPERO CRD42017065193) [40], and the meta-analysis was performed following the PRISMA (Preferred Reporting Items for the Systematic Reviews and Meta-analyses) statement, the PRISMA network statement, and the Cochrane Collaboration recommendations [41–43]. Randomized trials comparing at least two different treatment arms were searched using PubMed, EMbase and the Cochrane Library (see Supplementary Table 4 for more details). No language restrictions or filters were imposed. The searches were conducted from the databases’ inceptions to May 2017.

Inclusion criteria were as follows: randomized controlled trials of patients with gastrointestinal cancers (such as colorectal cancer, gastric cancer, etc.); patients of any age, gender, tumor stage, and histological grade; either PSK used individually for treatment or combined treatment with chemotherapy drugs. Exclusion criteria were as follows: review or pooled analyses of using PSK for gastrointestinal cancers; no relevant intervention in the studies; and only meeting abstracts could be found in databases. Moreover, studies of inappropriate tumor types (non GIC) were excluded, as were those with no control group, non-human studies and with no specific data.

### Data abstraction and assessment of risk of bias

Two investigators (MY and WXF) independently abstracted data on study, patients, and treatment related characteristics onto a standardized form; discrepancies were resolved by consensus, referring back to the original study, or in consultation with a third reviewer (MXJ). Data on efficacy and safety were abstracted from original studies. We extracted trial design, trial size, details of treatment arms including dose, period and duration of follow-up, type of outcome (efficacy and safety), and outcome data for each time-point of interest. Whenever necessary, we approximated means and measures of dispersion from figures in the original studies [44]. We extracted results from intention-to-treat analyses whenever possible.

The risk of bias of the individual studies was assessed using the Physiotherapy Evidence Database (PEDro) scale score [45]. The PEDro scale score is an 11-item scale that assesses the quality of RCTs. For instance, if the answer to the first item is “NO”, the study is excluded from the meta-analysis. When the PEDro score is greater than 4 (the max score was 10), the study is considered to be of high quality. The risk of bias was performed independently by two investigators and was resolved by a third when appropriate.

### Outcomes

We performed subgroup analyses and meta-regression according to the cancer types (CRC, EPC and GC) and treatment arms [PSK+CT vs CT alone (po), PSK+CT vs CT alone (iv+po), PSK+RT+CT vs RT+CT, PSK+RT vs RT+PSK vs CT alone (po), PSK plus 5-Fu vs 5-Fu alone, and PSK plus UFT vs UFT alone].

The primary efficacy outcome was 1-year to 7-year overall survival (OS) associated with PSK arm vs control arm, which could be stratified by cancer type and treatment arm. Our secondary efficacy outcome was 1-year to 7-year disease-free survival (DFS) associated with PSK arm vs control arm, which could be stratified by cancer type and treatment arms.

Our primary safety outcomes were gastrointestinal and hematologic toxicity; the former including nausea-vomiting, diarrhea, anorexia and obstipation and the latter including anemia, leukopenia and thrombocytopenia. Our secondary safety outcomes included abnormal hepatic function, abnormal pain and stomatitis.

### Data synthesis and statistical analysis

We defined studies reporting multiple treatments and controls as sub-studies (marked as a/b) to avoid double-counting and mistreating data. First, direct meta-analysis was performed with random-effects models because they are likely the most appropriate and conservative methodology to account for between-trial heterogeneity for each comparison [46–47]. To estimate pooled odds ratios (OR) and 95% confidence intervals (95% CI) incorporating heterogeneity was incorporated within and between studies, with STATA v14.0. Statistical heterogeneity was assessed with *P* values and *I*^*2*^ statistics, with values higher than 50% indicating substantial heterogeneity [48].We used Begg’s and Egger’s tests to detect publication bias [49].

Second, we conducted a random-effects network meta-analysis using STATA v14.0. We summarized the results of the network meta-analysis with OR and their credible intervals (CrI) [50]. A common heterogeneity parameter was assumed for all comparisons; we also assessed global heterogeneity using *P* values and the *I*^*2*^ statistic.

The relative efficacy and safety of each treatment resulted from the combination of the direct evidence between the two treatment arms and the indirect evidence derived from the network meta-analysis, which are assumed to be coherent [46]. Inconsistency between direct and indirect sources of evidence was statistically assessed globally (by comparison of the fit and parsimony of consistency and inconsistency models) and locally (by calculation of the difference between direct and indirect estimates in all closed loops in the network) [51]. When a direct connection between two treatment arms was not available, the result was obtained only from indirect evidence.

We estimated the ranking probabilities for all treatments of being at each possible rank for each treatment arm. The treatment hierarchy was summarized and reported as surface under the cumulative ranking curve (SUCRA) [52], ranging from 1, indicating that the treatment has a high likelihood of being best, to 0, indicating that the treatment has a high likelihood of being worst. A high SUCRA score corresponds to a higher ranking of survival rate from cancer compared with other treatments.

### Quality of evidence

We assessed the quality of evidence for our primary outcomes according to the Grading of Recommendations Assessment, Development and Evaluation (GRADE) system using GRADEpro GDT [53–54]. The GRADE system assesses risk of bias (study limitations), imprecision, inconsistency, indirectness of study results, and publication bias (classifying each as high, moderate, low, or very low) across the body of evidence to derive an overall summary of the quality of evidence.

### Patient involvement

No patients were involved in setting the research question or the outcome measures, nor were they involved in developing plans for design or implementation of the study. No patients were asked for advice on the interpretation or presentation of results. There are no plans to disseminate the results of the research to study participants or the relevant patient community.

## SUPPLEMENTARY MATERIALS FIGURES AND TABLES








