# Supplementary material for: Can polysaccharide K improve therapeutic efficacy and safety in gastrointestinal cancer? a systematic review and network meta-analysis
Source: Oncotarget. 2017 Jul 6;8(51):89108–18. doi: 10.18632/oncotarget.19059 (PMC5687673; doi:10.18632/oncotarget.19059)
Supplement: Supplementary file 1 [file oncotarget-08-89108-s001.pdf]

Can polysaccharide K improve therapeutic efficacy and safety in gastrointestinal cancer? a systematic review and network meta-analysis

Supplementary Materials

Supplementary Table 1A: Monosaccharide composition in sugar portion of PSK

Monosaccharide	Composition (%)
Glucose	74.6
Galactose.	2.7
Mannose	15.5
Xylose	4.8
Fucose	2.4

Supplementary Table 1B: Amino acid composition in the protein portion of PSK

Amino Acid	Composition (%)	Amino Acid	Composition (%)
Aspartic acid	13.2	Methionine	1.9
Threonine	4.5	Isoleucine	5.9
Serine	4.7	Leucine	13.4
Glutamic acid	14.4	Tyrosine	2.9
Proline	+	Phenylalanine	6.7
Glycine	7.8	Tryptophan	+
Alanine	9.2	Lysine	2.8
Cystine	+	Histidine	2.3
Valine	9.6	Arginine	0.7

Supplementary Table 2: Study characteristics. See Supplementary_Table_2

Supplementary Table 3: PEDro scale score. See Supplementary_Table_3

Supplementary Table 4: Search strategies for pubmed, EMBASE and the cochrane library database

Search strategies for PubMed
#1. (protein-bound[All Fields] AND ("krestin"[Supplementary Concept] OR "krestin"[All Fields] OR "polysaccharide k"[All Fields]) AND ("gastrointestinal neoplasms"[MeSH Terms] OR ("gastrointestinal"[All Fields] AND "neoplasms"[All Fields]) OR "gastrointestinal neoplasms"[All Fields] OR ("gastrointestinal"[All Fields] AND "cancer"[All Fields]) OR "gastrointestinal cancer"[All Fields])) AND Clinical Trial[ptyp]
#2. (protein-bound[All Fields] AND ("krestin"[Supplementary Concept] OR "krestin"[All Fields] OR "polysaccharide k"[All Fields]) AND ("colorectal neoplasms"[MeSH Terms] OR ("colorectal"[All Fields] AND "neoplasms"[All Fields]) OR "colorectal neoplasms"[All Fields] OR ("colorectal"[All Fields] AND "cancer"[All Fields]) OR "colorectal cancer"[All Fields])) AND Clinical Trial[ptyp]
#3.(protein-bound[All Fields] AND ("krestin"[Supplementary Concept] OR "krestin"[All Fields] OR "polysaccharide k"[All Fields]) AND ("oesophagus cancer"[All Fields] OR "esophageal neoplasms"[MeSH Terms] OR ("esophageal"[All Fields] AND "neoplasms"[All Fields]) OR "esophageal neoplasms"[All Fields] OR ("esophagus"[All Fields] AND "cancer"[All Fields]) OR "esophagus cancer"[All Fields])) AND Clinical Trial[ptyp]
#4. (protein-bound[All Fields] AND ("krestin"[Supplementary Concept] OR "krestin"[All Fields] OR "polysaccharide k"[All Fields]) AND ("stomach neoplasms"[MeSH Terms] OR ("stomach"[All Fields] AND "neoplasms"[All Fields]) OR "stomach neoplasms"[All Fields] OR ("gastric"[All Fields] AND "cancer"[All Fields]) OR "gastric cancer"[All Fields])) AND Clinical Trial[ptyp]
#5.("glucuronosyltransferase"[MeSH Terms] OR "glucuronosyltransferase"[All Fields] OR ("udp"[All Fields] AND "glucuronosyltransferase"[All Fields]) OR "udp glucuronosyltransferase"[All Fields]) AND 1a7[All Fields] AND ("colorectal neoplasms"[MeSH Terms] OR ("colorectal"[All Fields] AND "neoplasms"[All Fields]) OR "colorectal neoplasms"[All Fields] OR ("colorectal"[All Fields] AND "carcinoma"[All Fields]) OR "colorectal carcinoma"[All Fields])
#6. #1and #2 and#3 and#4 and#5
**Search strategies for EMbase**
#1 'protein-bound polysaccharide K$':ab,ti
#2 'PSK$':ab,ti
#3 'gastrointestinal cancer$':ab,ti
#4 'colorectal cancer$'/exp
#5 'esophageal cancer$':ab,ti
#6 'gastric cancer':ab,ti
#7. #1and #2 and#3 and#4 and#5 and #6
**Search strategies for Cochrane library**
#1.protein-bound polysaccharide K and PSK, gastrointestinal cancer or colorectal cancer or esophageal cancer or gastric cancer.
#2. MeSH descriptor

Supplementary Table 5: Meta-analysis and meta-regression for the 1-year to 7-year overall survivals(OSs) and disease-free survivals(DFSs) associated with the PSK arm vs the control arm.
See Supplementary_Table_5
