# Supplementary material for: Can polysaccharide K improve therapeutic efficacy and safety in gastrointestinal cancer? a systematic review and network meta-analysis
Source: Oncotarget. 2017 Jul 6;8(51):89108–18. doi: 10.18632/oncotarget.19059 (PMC5687673; doi:10.18632/oncotarget.19059)
Supplement: Supplementary file 2 [file oncotarget-08-89108-s002.doc]

**Supplementary Table 2 Study Characteristics.**

| **Publication, Year, Location** | **Subjects** | | | | | **Design** | | |  |
| --- | --- | --- | --- | --- | --- | --- | --- | --- | --- |
|  | **Study design** | **Participants(N,age,male%)** | **Cancer type** | **Tumor stage(I-II/III-IV)** | **Histological grade (well/moderate/poor)** | **PSK Arm** | **Control Arm** | **Treatment period** | **Follow-up** |
| Ito K, 2004, Japan [12] | RCT | P:220; 60.4; 55.9%  C:221; 60.0; 54.8 | CRC | - | P:110/96/14  C:115/95/11 | PSK(3g/day), orally + control | CT: oral 5-FU (200 mg per day) | 4 weeks | 7 years |
| Mitomi T , 1992,CRC[13] | RCT | P(a):124;-;58.06%  C(a):125;-;52%  P(b):97;-;54.64%  C(b):102;-;43.14% | CRC  a:Colon cancer  b: Rectum cancer | P(a):2/122  C(a):1/124  P(b):5/92  C(b):4/98 | P(a):79/39/2  P(b):64/53/3  C(a):49/40/4  C(b):52/39/2 | PSK(3g/day), orally + control | CT: Oral 5-FU(200mg/day) | P:PSK orally over 3 years+6 months  C: 6 months | 5 years |
| Ohwada S, 2003, Japan[14] | RCT | P:134; -; 52.2%  C:67; -; 62.7% | CRC(colon and rectum) | P:35/99  C:20/47 | P:123/11  C:66/1 | PSK(3g/day), orally + control | CT: Oral UFT(300 mg/day) | More than 2 years | 5 years |
| Ohwada S, 2004, Japan[15] | RCT | P:137; -; 51.8%  C:68; -; 63.2% | CRC(colon and rectum) | P:12/125  C:11/57 | P:45/81/11  C:36/31/1 | PSK(3g/day), orally + control | CT: Oral UFT(300 mg/day) | More than 2 years | 5 years |
| Ohwada S, 2006, Japan[16] | RCT | P:137; -; 51.8%  C:68; -; 63.2% | CRC(colon and rectum) | P:55/39  C:25/22 | P:34/59/1  C:26/21/0 | PSK(3g/day), orally + control | CT: Oral UFT(300 mg/day) | More than 2 years | 5 years |
| Sadahiro S,2010,Japan[17] | RCT | P;:15,60±10,80%  C:15,65±6,73.33% | CRC(rectum) | - | P:6/9  C:6/9: | PSK(3g/day), orally + control | CT: Oral S-1 80 mg/m2/day | 4weeks | - |
| Takahashi Y, 2005, Japan[18] | RCT | P:43,61.2, 55.8%  C:44,59.5, 58.5% | CRC(colon ) | - | P:25/16/2  C:18/21/2 | intravenous infusion of 5-FU (1,000 mg/m2/24 h x 2) once a week for 3 to 4 weeks. From week 4, PSK (300 mg/day) was given for 4 weeks followed by 5-FU (200 mg/day) for 4 weeks to the PSK group | CT: 10 courses of intermittent treatment with 5-FU | 8 weeks | 7 years |
| Torisu M, 1990, Japan[19] | RCT | P:56,59.3,-  C:55,58.4,- | CRC | - | P:37/8/3  C:39/7/3 | PSK(3g/day) for 2 months, then 2 g daily until 24 months and 1 g daily thereafter | Placebo | More than 2 years | 7 years |
| Yamashita K, 2007, Japan[20] | RCT | P:99;-,55.56%  C:103,-,53.40% | CRC | P:57/42  C:55/58 | - | PSK + control | CT: 5-FU | 2months | 5 years |
| Ogoshi K, 1995, Japan[21] | RCT | P(a): 56,59,85.71%  P(b):38,58.5,89.47%  C(a):49,57,80.70%  C(b):31,65,83.87% | EPC | P(a):15/36  P(b):16/20  C(a):9/38  C(b):12/18 | P(a):16/27/10  P(b):14/15/2  C(a):13/24/3  C(b):15/9/3 | PSK(3g/day), +control  P(a): PSK+RT+CT  P(b):PSK+RT | RT:2.0 Gy daily  CT:5-FU 600mg/d  C(a):RT+CT  C(b):RT | 3months | 5 years |
| Ogoshi K, 2009, Japan[22] | RCT | P(a): 49,59,89.80%  P(b):36,58.5,91.67%  C(a):41,57,92.69%  C(b):32,65,87.5% | EPC | P(a):15/31  P(b):15/20  C(a):7/32  C(b):14/17 | P(a):16/24/7  P(b):12/16/2  C(a):12/18/4  C(b):12/14/7 | P(a):PSK(3g/day), orally +RT+CT  P(b):PSK(3g/day), orally+RT  PSK(3g/day), orally | C(a):RT:2.0 Gy daily  C(b):CT:5-FU 600mg/d, orally | 3months | 5 years |
| Ahn MS, 2013,Korea[23] | RCT | P(a): 44,55,65.9%  P(b):38,49,57.9% | GC | P(a):19/25  P(b):16/22 | - | P(a):PSK(3g/day), orally + 5-FU (500 mg/m2) i.v.  P(b):PSK(3g/day), orally+ UFT p.o | - | P(a):4 months  P(b):12 months | 10 years |
| Akagi J, 2010 Japan[24] | RCT | P:10,67.5±3.9,80%  C:11,69.7±9.4,54.55% | GC | P:2/8  C:1/10 | - | P:PSK(3g/day), orally+control | C:CT: UFT(300m g/day) p.o. | Less than 2 years | 3 years |
| Hattori T, 1990, Japan[25] | RCT | P(a): 1426  P(b):1338  C(a):1357%  C(b):1363 | GC | - | - | PSK(3g/day), orally+control  P(a): PSK+C(a)  P(b):PSK+C(b) | C(a):CT:Tegafur: 600 mg/day,p.o.  C(b):CT+0K-432:Tegafur+:0.5~5.0 KE/day | 8 months | 3 years |
| Kondo T, 1991, Japan[26] | RCT | P:49,-,63.27%  C:54,-,59.26% | GC | P:18/31  C:22/32 | - | PSK(2mg/m^2^/day), orally+control | C:CT:Carbazilquinone 2mg/m^2^/day, i,v.+p.o. | 4 weeks | 7 years |
| Kono K, 2008, Japan[27] | RCT | P:10,69±18,70%  C:10,67±12,80% | GC | - | - | PSK(3g/day), orally+control | C:CT: Oral S-1 80 mg/m2/day | 16 weeks | - |
| Maehara Y, 1990, Japan[28] | RCT | P:118  C:137 | GC | - | - | PSK(3g/day), p.o. | C:CT:Tegafur 600 mg/day | 1 year | 15 years |
| Nakazato H , 1994, Japan[29] | RCT | P:124,58,66.93%  C:129,59,66.67% | GC | P:44/80  C:45/84 | P:22/32/48  C:18/28/62 | PSK(3g/day)+control | C:CT:5-Fu(150mg/day),po | 4 weeks | 5 years |
| Niimoto M, 1988,Japan[30] | RCT | P(a): 189  P(b):191  C:199 | GC | - | - | P(a): PSK(3g/day)  P(b):PSK(3g/day) +futraful(600rag/day p.o. ) | C:CT: futraful(600rag/day p.o. ) | 1 year | 5 years |
| Saji S, 1999,Japan[31] | RCT | P:124  C:129 | GC | - | - | PSK(3g/day), orally+control | C:CT:MMC+5-Fu.i.v+p.o. | 2 year | 7 year |
| Sakamoto J,1992,Japan[32] | RCT | P:100  C:118 | GC | - | - | PSK(3g/day), orally + control | CT: Oral 5-FU(150mg/day) | 2weeks-19months | 4years |
| Toge T, 2000, Japan[33] | RCT | P(a):195,55.6±11.7,59%  P(b):182,56.1±11.7,34%  C(a):192,57.5±11.3,62.4%  C(b):182,56.5±12.4,62.6% | GC | P(a):116/72  P(b):113/67  C(a):124/65  C(b):102/75 | - | PSK(3g/day), orally+control | CT: MMC (20+10 mg/day iv) plus FT(600-800 mg/day po), iv+po | - | 5 years |
| Nio Y, 1992, Japan[34] | RCT | P(a):22,61.2±11.9,40.91%  P(b):11,60.5±12.0,54.55%  C:14,63.7±11.2,64.29% | GIC: GC and CRC | P(a):10/10  P(b):4/5  C:6/6 | P(a):7/3/5  P(b):2/3/5  C:4/5/4 | PSK(3g/day), orally | Placebo | P(a): less than 14days  P(b): more than 14days | - |

C, control group; CRC,colorectal cancer CT,chemotherapy; DFS,disease free survival; EPC,Esophageal Cancer; GC,gastric cancer; GIC,gastrointestinal cancer; IT, immunochemotherapy; MMC,mitomycin C; OS,overall survival; P, PSK group; PSK, Polysaccharide K; RCT, randomized controlled trails; RT，radiotherapy; UFT, Tegafur/uracil.
