# Supplementary material for: Can polysaccharide K improve therapeutic efficacy and safety in gastrointestinal cancer? a systematic review and network meta-analysis
Source: Oncotarget. 2017 Jul 6;8(51):89108–18. doi: 10.18632/oncotarget.19059 (PMC5687673; doi:10.18632/oncotarget.19059)
Supplement: Supplementary file 3 [file oncotarget-08-89108-s003.docx]

**Supplementary Table 3 PEDro scale score.**

| Publication(year) | Eligibility criteria specified(Yes/No) | Random allocation(0/1) | Concealed allocation(0/1) | Comparable at baseline(0/1) | Blinded subjects(0/1) | Blinded therapists(0/1) | Blinded assessors(0/1) | Adequate follow-up(0/1) | Intention-to-treat analysis(0/1) | Between group comparisons(0/1) | Point estimates and variability(0/1) | **Summary** |
| --- | --- | --- | --- | --- | --- | --- | --- | --- | --- | --- | --- | --- |
| Ito K, 2004, Japan [12] | Yes | 1 | 0 | 1 | 0 | 0 | 1 | 1 | 1 | 1 | 1 | 7 |
| Mitomi T , 1992,CRC[13] | Yes | 1 | 0 | 1 | 0 | 0 | 0 | 1 | 0 | 1 | 1 | 5 |
| Ohwada S, 2003, Japan[14] | Yes | 1 | 0 | 1 | 1 | 1 | 0 | 1 | 0 | 1 | 1 | 7 |
| Ohwada S, 2004, Japan[15] | Yes | 1 | 0 | 1 | 1 | 1 | 0 | 1 | 0 | 1 | 1 | 7 |
| Ohwada S, 2006, Japan[16] | Yes | 1 | 0 | 1 | 0 | 0 | 0 | 1 | 0 | 1 | 1 | 5 |
| Sadahiro S,2010,Japan[17] | Yes | 1 | 1 | 1 | 0 | 0 | 0 | 0 | 0 | 1 | 1 | 5 |
| Takahashi Y, 2005, Japan[18] | Yes | 1 | 0 | 1 | 0 | 0 | 0 | 1 | 1 | 1 | 1 | 6 |
| Torisu M, 1990, Japan[19] | Yes | 1 | 0 | 1 | 1 | 1 | 0 | 1 | 0 | 1 | 1 | 7 |
| Yamashita K, 2007, Japan[20] | Yes | 1 | 0 | 1 | 1 | 1 | 0 | 0 | 0 | 1 | 1 | 6 |
| Ogoshi K, 1995, Japan[21] | Yes | 1 | 0 | 1 | 0 | 0 | 0 | 1 | 0 | 1 | 1 | 6 |
| Ogoshi K, 2009, Japan[22] | Yes | 1 | 0 | 1 | 0 | 0 | 0 | 1 | 0 | 1 | 1 | 6 |
| Ahn MS, 2013,Korea[23] | Yes | 1 | 0 | 1 | 0 | 0 | 0 | 1 | 1 | 1 | 1 | 7 |
| Akagi J, 2010 Japan[24] | Yes | 1 | 0 | 1 | 0 | 0 | 0 | 1 | 1 | 1 | 1 | 6 |
| Hattori T, 1990, Japan[25] | Yes | 1 | 0 | 1 | 0 | 0 | 0 | 1 | 0 | 1 | 1 | 5 |
| Kondo T, 1991, Japan[26] | Yes | 1 | 0 | 1 | 0 | 0 | 0 | 1 | 0 | 1 | 1 | 5 |
| Kono K, 2008, Japan[27] | Yes | 1 | 0 | 1 | 0 | 0 | 0 | 0 | 0 | 1 | 1 | 4 |
| Maehara Y, 1990, Japan[28] | Yes | 1 | 0 | 1 | 0 | 0 | 0 | 1 | 0 | 1 | 1 | 5 |
| Nakazato H , 1994, Japan[29] | Yes | 1 | 0 | 1 | 0 | 0 | 0 | 1 | 0 | 1 | 1 | 5 |
| Niimoto M, 1988,Japan[30] | Yes | 1 | 0 | 1 | 0 | 0 | 0 | 1 | 0 | 1 | 1 | 5 |
| Saji S, 1999,Japan[31] | Yes | 1 | 0 | 1 | 0 | 0 | 0 | 1 | 0 | 1 | 1 | 5 |
| Sakamoto J,1992,Japan[32] | Yes | 1 | 0 | 1 | 0 | 0 | 0 | 1 | 0 | 1 | 1 | 5 |
| Toge T, 2000, Japan[33] | Yes | 1 | 0 | 1 | 0 | 0 | 0 | 1 | 0 | 1 | 1 | 5 |
| Nio Y, 1992, Japan[34] | Yes | 1 | 0 | 1 | 0 | 0 | 0 | 1 | 0 | 1 | 1 | 5 |

0, indicates the criterion was not satisfied; 1,the criterion was satisfied.
