# Supplementary material for: Can polysaccharide K improve therapeutic efficacy and safety in gastrointestinal cancer? a systematic review and network meta-analysis
Source: Oncotarget. 2017 Jul 6;8(51):89108–18. doi: 10.18632/oncotarget.19059 (PMC5687673; doi:10.18632/oncotarget.19059)
Supplement: Supplementary file 4 [file oncotarget-08-89108-s004.doc]

**Supplementary Table 5: Meta-analysis and meta-regression for the 1-year to 7-year overall survivals(OSs) and disease-free survivals(DFSs) associated with the PSK arm vs the control arm**.

| **Outcome** | **Patients(P/C)** | **OR(95%CI)** | **Heterogeneity(*P*,*I*^2^)** | **Meta-regression(*P*)** | **Quality of evidence** | **Publication bias** | |
| --- | --- | --- | --- | --- | --- | --- | --- |
|  |  |  |  |  |  | **Begg’s(*P*)** | **Egger’s(*P*)** |
| **Overall survival (OS)** |  |  |  |  |  |  |  |
| ***1-year OS*** |  |  |  |  |  |  |  |
| Total | 22(2406/2233) | 1.59(1.12, 2.25)* | *P*=0.012, *I*^2^=45.8% |  | High | *P*=0.057 | *P*=0.112 |
| Cancer type |  |  |  |  |  |  |  |
| CRC | 9(1047/853) | 3.54(1.69, 7.40)* | *P*=0.318, *I*^2^=14.3% | *P*=0.000^†^ | High | *P*=0.621 | *P*=0.429 |
| EPC | 4(179/153) | 1.73(1.06, 2.81)* | *P*=0.424, *I*^2^=0.0% |  | High | *P*=0.174 | *P*=0.481 |
| GC | 9(1077/1180) | 1.16(0.73, 1.82) | *P*=0.026, *I*^2^=54.2%^#^ |  | Moderate | *P*=0.835 | *P*=0.838 |
| Treatment arms |  |  |  |  |  |  |  |
| PSK+CT vs CT alone(po) | 10(1273/1093) | 2.19(1.24,3.89)* | *P*=0.037, *I*^2^=48.1% | *P*=0.006^†^ | High | *P*=0.161 | *P*=0.058 |
| PSK+CT vs CT alone(iv+po) | 5(591/596) | 1.43(0.92, 2.23) | *P*=0.540, *I*^2^=0.0% |  | High | *P*=0.624 | *P*=0.806 |
| PSK+RT+CT vs RT+CT | 2(105/90) | 2.27(1.20, 4.26)* | *P*=0.492, *I*^2^=0.0% |  | Moderate | *P*=0.317 | *P*=0.429 |
| PSK+RT vs RT | 2(74/63) | 1.17(0.55,2.50) | *P*=0.437, *I*^2^=0.0% |  | Moderate | *P*=0.317 | *P*=1.000 |
| PSK vs CT alone(po) | 2(307/336) | 0.61(0.16, 2.26) | *P*=0.020, *I*^2^=81.6%^#^ |  | Low | *P*=0.317 | *P*=1.000 |
| Sub treatment -PSK+CT vs CT alone(po) |  |  |  |  |  |  |  |
| PSK plus 5-Fu vs 5-Fu alone | 5(664/680) | 1.14(0.55, 2.34) | *P*=0.446, *I*^2^=0.0% | *P*=0.013^†^ | High | *P*=0.086 | *P*=0.024^‡^ |
| PSK plus UFT vs UFT alone | 4(418/214) | 8.03(3.17, 20.35)* | *P*=0.735, *I*^2^=0.0% |  | High | *P*=1.000 | *P*=0.577 |
|  |  |  |  |  |  |  |  |
| ***2-year OS*** |  |  |  |  |  |  |  |
| Total | 22(2406/2233) | 1.43(1.17, 1.75)* | *P*=0.083, *I*^2^=31.0% |  | High | *P*=0.866 | *P*=0.441 |
| Cancer type |  |  |  |  |  |  |  |
| CRC | 9(1047/853) | 1.60(1.13, 2.28)* | *P*=0.331, *I*^2^=12.5% | *P*=0.000^†^ | High | *P*=1.000 | *P*=0.145 |
| EPC | 4(179/153) | 1.22(0.79, 1.89) | *P*=0.405, *I*^2^=0.0% |  | High | *P*=0.497 | *P*=0.149 |
| GC | 9(1077/1180) | 1.42(1.04, 1.93)* | *P*=0.025, *I*^2^=54.4%^#^ |  | Moderate | *P*=0.835 | *P*=0.915 |
| Treatment arms |  |  |  |  |  |  |  |
| PSK+CT vs CT alone(po) | 10(1273/1093) | 1.75(1.38, 2.27)* | *P*=0.732, *I*^2^=0.0% | *P*=0.004^†^ | High | *P*=0.788 | *P*=0.351 |
| PSK+CT vs CT alone(iv+po) | 5(591/596) | 1.64(1.22, 2.22)* | *P*=0.444, *I*^2^=0.0% |  | High | *P*=0.327 | *P*=0.292 |
| PSK vs placebo | 1(56/55) | 0.67 (0.24,1.91) | *-* |  | - | - | - |
| PSK+RT+CT vs RT+CT | 2(105/90) | 1.66 (0.94, 2.95) | *P*=0.692, *I*^2^=0.0% |  | Moderate | *P*=0.317 | *P*=1.000 |
| PSK+RT vs RT | 2(74/63) | 0.79(0.40,1.56) | *P*=0.788, *I*^2^=0.0% |  | Moderate | *P*=0.317 | *P*=1.000 |
| PSK vs CT alone(po) | 2(307/336) | 0.87(0.31,2.42) | *P*=0.006, *I*^2^=86.8%^#^ |  | Low | *P*=0.317 | *P*=1.000 |
| Sub treatment -PSK+CT vs CT alone(po) |  |  |  |  |  |  |  |
| PSK plus 5-Fu vs 5-Fu alone | 5(664/680) | 1.52(1.04, 2.22)* | *P*=0.449, *I*^2^=0.0% | *P*=0.054 | High | *P*=0.142 | *P*=0.020 |
| PSK plus UFT vs UFT alone | 4(418/214) | 1.84(1.13,3.01)* | *P*=0.723, *I*^2^=0.0% |  | High | *P*=0.497 | *P*=0.985 |
|  |  |  |  |  |  |  |  |
| ***3-year OS*** |  |  |  |  |  |  |  |
| Total | 24(5170/4953) | 1.35(1.14,1.59)* | *P*=0.002, *I*^2^=51.5%^#^ |  | Moderate | *P*=0.573 | *P*=0.468 |
| Cancer type |  |  |  |  |  |  |  |
| CRC | 9(1047/853) | 1.72(1.15,2.56)* | *P*=0.449, *I*^2^=0.0% | *P*=0.000^†^ | High | *P*=0.211 | *P*=0.089 |
| EPC | 4(179/153) | 1.22(0.79,1.89) | *P*=0.805, *I*^2^=0.0% |  | High | *P*=0.174 | *P*=0.116 |
| GC | 11(3944/3947) | 1.24(1.03,1.51)* | *P*=0.006, *I*^2^=59.3%^#^ |  | Moderate | *P*=0.835 | *P*=0.997 |
| Treatment arms |  |  |  |  |  |  |  |
| PSK+CT vs CT alone(po) | 11(2699/2450) | 1.59(1.21,2.10)* | *P*=0.019, *I*^2^=52.9%^#^ | *P*=0.014^†^ | Moderate | *P*=0.392 | *P*=0.010^‡^ |
| PSK+CT vs CT alone(iv+po) | 5(591/596) | 1.59(1.02,2.48)* | *P*=0.066, *I*^2^=54.7%^#^ |  | Moderate | *P*=0.624 | *P*=0.608 |
| PSK vs placebo | 1(56/55) | 0.81(0.37,1.77) | - |  | - |  |  |
| PSK+RT+CT vs RT+CT | 2(105/90) | 1.47(0.82,2.62) | *P*=0.849, *I*^2^=0.0% |  | Moderate | *P*=0.317 | *P*=1.000 |
| PSK+RT vs RT | 2(74/63) | 0.56(0.48,1.87) | *P*=0.864, *I*^2^=0.0% |  | Moderate | *P*=0.317 | *P*=1.000 |
| PSK vs CT alone(po) | 2(307/336) | 0.90(0.38,2.14) | *P*=0.013, *I*^2^=83.7%^#^ |  | Low | *P*=0.174 | *P*=0.116 |
| PSK+CT+IT vs CT+CT(po) | 1(1338/1363) | 1.02(0.87,1.20) | - |  | - |  |  |
| Sub treatment -PSK+CT vs CT alone(po) |  |  |  |  |  |  |  |
| PSK plus 5-Fu vs 5-Fu alone | 5(664/680) | 1.58(1.06, 2.36)* | *P*=0.226, *I*^2^=29.3% | *P*=0.251 | High | *P*=0.142 | *P*=0.010^‡^ |
| PSK plus UFT vs UFT alone | 4(418/214) | 2.02(1.15,3.56)* | *P*=0.209, *I*^2^=33.9% |  | High | *P*=0.497 | *P*=0.631 |
|  |  |  |  |  |  |  |  |
| ***4-year OS*** |  |  |  |  |  |  |  |
| Total | 21(2396/2222) | 1.41(1.15, 1.73)* | *P*=0.003, *I*^2^=52.3%^#^ |  | Moderate | *P*=0.526 | *P*=0.730 |
| Cancer type |  |  |  |  |  |  |  |
| CRC | 9(1047/853) | 1.55(1.00, 2.41)* | *P*=0.001, *I*^2^=68.7%^#^ | *P*=0.016^†^ | Moderate | *P*=0.532 | *P*=0.571 |
| EPC | 4(179/153) | 1.24(0.79, 1.95) | *P*=0.864, *I*^2^=0.0% |  | High | *P*=0.497 | *P*=0.176 |
| GC | 8(1170/1216) | 1.34(1.04, 1.72)* | *P*=0.052, *I*^2^=49.9% |  | High | *P*=1.000 | *P*=0.691 |
| Treatment arms |  |  |  |  |  |  |  |
| PSK+CT vs CT alone(po) | 9(1263/1082) | 1.65(1.16, 2.34)* | *P*=0.006, *I*^2^=62.9%^#^ | *P*=0.003^†^ | Moderate | *P*=0.061 | *P*=0.076 |
| PSK+CT vs CT alone(iv+po) | 5(591/596) | 1.54(1.12,2.13)* | *P*=0.232, *I*^2^=28.4% |  | High | *P*=0.624 | *P*=0.806 |
| PSK vs placebo | 1(56/55) | 0.77(0.36,1.63) | - |  | - |  |  |
| PSK+RT+CT vs RT+CT | 2(105/90) | 1.45(0.79,2.66) | *P*=0.944, *I*^2^=0.0% |  | Moderate | *P*=0.317 | *P*=1.000 |
| PSK+RT vs RT | 2(74/63) | 1.01(0.51,2.00) | *P*=0.716, *I*^2^=0.0% |  | Moderate | *P*=0.317 | *P*=1.000 |
| PSK vs CT alone(po) | 2(307/338) | 0.93(0.51,1.69) | *P*=0.074, *I*^2^=68.7%^#^ |  | Low | *P*=0.317 | *P*=1.000 |
| Sub treatment -PSK+CT vs CT alone(po) |  |  |  |  |  |  |  |
| PSK plus 5-Fu vs 5-Fu alone | 5(664/680) | 1.25(0.77,2.02) | *P*=0.030, *I*^2^=62.8%^#^ | *P*=0.809 | Moderate | *P*=1.000 | *P*=0.189 |
| PSK plus UFT vs UFT alone | 3(408/203) | 2.69(1.62,4.47)* | *P*=0.218, *I*^2^=34.3% |  | High | *P*=0.602 | *P*=0.777 |
|  |  |  |  |  |  |  |  |
| ***5-year OS*** |  |  |  |  |  |  |  |
| Total | 21(2396/2222) | 1.37(1.12,1.68)* | *P*=0.001, *I*^2^=55.3%^#^ |  | Moderate | *P*=0.877 | *P*=0.083 |
| Cancer type |  |  |  |  |  |  |  |
| CRC | 9(1047/853) | 1.47(0.97,2.22) | *P*=0.001, *I*^2^=70.4%^#^ | *P*=0.550 | Moderate | *P*=0.835 | *P*=0.538 |
| EPC | 4(179/153) | 1.24(0.79,1.95) | *P*=0.864, *I*^2^=0.0% |  | High | *P*=0.497 | *P*=0.176 |
| GC | 8(1170/1216) | 1.32(1.01,1.72)* | *P*=0.027, *I*^2^=55.8%^#^ |  | Moderate | *P*=0.805 | *P*=0.819 |
| Treatment arms |  |  |  |  |  |  |  |
| PSK+CT vs CT alone(po) | 9(1263/1082) | 1.62(1.33,1.98)* | *P*=0.367, *I*^2^=8.2% | *P*=0.076 | High | *P*=0.404 | *P*=0.586 |
| PSK+CT vs CT alone(iv+po) | 5(591/596) | 1.58(1.05,2.38)* | *P*=0.052, *I*^2^=57.4%^#^ |  | Moderate | *P*=0.624 | *P*=0.705 |
| PSK vs placebo | 1(56/55) | 0.21(0.09,0.50) | - |  | - |  |  |
| PSK+RT+CT vs RT+CT | 2(105/90) | 1.45(0.79,2.66) | *P*=0.944, *I*^2^=0.0% |  | Moderate | *P*=0.317 | *P*=1.000 |
| PSK+RT vs RT | 2(74/63) | 1.01(0.51,2.00) | *P*=0.716, *I*^2^=0.0% |  | Moderate | *P*=0.317 | *P*=1.000 |
| PSK vs CT alone(po) | 2(307/338) | 1.04(0.71,1.52) | *P*=0.238, *I*^2^=28.3% |  | Moderate | *P*=0.317 | *P*=1.000 |
| Sub treatment -PSK+CT vs CT alone(po) |  |  |  |  |  |  |  |
| PSK plus 5-Fu vs 5-Fu alone | 5(664/680) | 1.35(1.04,1.74)* | *P*=0.586, *I*^2^=0.0% | *P*=0.883 | High | *P*=0.624 | *P*=0.893 |
| PSK plus UFT vs UFT alone | 3(408/203) | 2.30(1.56,3.38)* | *P*=0.732, *I*^2^=0.0% |  | High | *P*=0.602 | *P*=0.824 |
|  |  |  |  |  |  |  |  |
| ***6-year OS*** |  |  |  |  |  |  |  |
| Total | 7(732/764) | 1.14(0.73,1.79) | *P*=0.002, *I*^2^=71.3%^#^ |  | Moderate | *P*=0.393 | *P*=0.993 |
| Cancer type |  |  |  |  |  |  |  |
| CRC | 3(319/320) | 1.10(0.43,2.84) | *P*=0.010, *I*^2^=78.5%^#^ | *P*=0.891 | Moderate | *P*=0.602 | *P*=0.947 |
| GC | 4(413/444) | 1.18(0.66,2.09) | *P*=0.009, *I*^2^=74.2%^#^ |  | Moderate | *P*=0.497 | *P*=0.931 |
| Treatment arms |  |  |  |  |  |  |  |
| PSK plus 5-Fu vs 5-Fu alone(po) | 2(344/350) | 1.39(0.97,1.98) | *P*=0.995, *I*^2^=0.0% | *P*=0.178 | High | *P*=0.317 | - |
| PSK+CT vs CT alone(iv+po) | 3(214/222) | 1.78(1.02,3.12)* | *P*=0.211, *I*^2^=35.7% |  | High | *P*=0.602 | *P*=0.881 |
| PSK vs placebo | 1(56/55) | 0.41(0.18,0.90) | - |  | - | - | - |
| PSK vs CT alone(po) | 1(118/137) | 0.62(0.37,1.03) | - |  | - | - | - |
|  |  |  |  |  |  |  |  |
| ***7-year OS*** |  |  |  |  |  |  |  |
| Total | 7(732/764) | 1.35(0.93,1.94) | *P*=0.027, *I*^2^=57.9%^#^ |  |  |  |  |
| Cancer type |  |  |  |  |  |  |  |
| CRC | 3(319/320) | 1.20(0.43,2.84) | *P*=0.007, *I*^2^=79.8%^#^ | *P*=0.660 | Moderate | *P*=0.602 | *P*=0.991 |
| GC | 4(413/444) | 1.18(0.66,2.09) | *P*=0.298, *I*^2^=18.5% |  | High | *P*=0.497 | *P*=0.949 |
| Treatment arms |  |  |  |  |  |  |  |
| PSK plus 5-Fu vs 5-Fu alone(po) | 2(344/350) | 1.46(1.03,2.08)* | *P*=0.821, *I*^2^=0.0% | *P*=0.540 | Moderate | *P*=0.317 | - |
| PSK+CT vs CT alone(iv+po) | 3(214/222) | 1.97(1.20,3.24)* | *P*=0.277, *I*^2^=22.0% |  | High | *P*=0.602 | *P*=0.979 |
| PSK vs placebo | 1(56/55) | 0.43(0.19,0.96) | - |  | - | - | - |
| PSK vs CT alone(po) | 1(118/137) | 1.11(0.68,1.82) | - |  | - | - | - |
|  |  |  |  |  |  |  |  |
| **Disease-free survival (DFS)** |  |  |  |  |  |  |  |
| ***1-year DFS*** |  |  |  |  |  |  |  |
| Total | 11(1216/1035) | 1.37(1.04,1.81)* | *P*=0.488, *I*^2^=0.0% |  | High | *P*=0.755 | *P*=0.811 |
| Cancer type |  |  |  |  |  |  |  |
| CRC | 8(948/750) | 1.46(1.05,2.04)* | *P*=0.472, *I*^2^=0.0% | *P*=0.533 | High | *P*=0.458 | *P*=0.321 |
| GC | 3(268/285) | 1.18(0.67,2.09) | *P*=0.294, *I*^2^=18.3% |  | High | *P*=0.602 | *P*=0.404 |
| Treatment arms |  |  |  |  |  |  |  |
| PSK plus 5-Fu vs 5-Fu alone | 5(665/695) | 1.21(0.83,1.76) | *P*=0.318, *I*^2^=15.1% | *P*=0.333 | High | *P*=0.117 | *P*=0.412 |
| PSK plus UFT vs UFT alone | 3(408/203) | 2.01(1.15,3.49)* | *P*=0.874, *I*^2^=0.0% |  | High | *P*=0.624 | *P*=0.759 |
|  |  |  |  |  |  |  |  |
| ***2-year DFS*** |  |  |  |  |  |  |  |
| Total | 11(1216/1035) | 1.68(1.36,2.07)* | *P*=0.804, *I*^2^=0.0% |  | High | *P*=0.399 | *P*=0.675 |
| Cancer type |  |  |  |  |  |  |  |
| CRC | 8(948/750) | 1.77(1.39,2.26)* | *P*=0.472, *I*^2^=0.0% | *P*=0.411 | High | *P*=0.322 | *P*=0.259 |
| GC | 3(268/285) | 1.44(0.96,2.16) | *P*=0.294, *I*^2^=18.3% |  | High | *P*=0.602 | *P*=0.656 |
| Treatment arms |  |  |  |  |  |  |  |
| PSK plus 5-Fu vs 5-Fu alone | 5(665/695) | 1.51(1.16,1.97)* | *P*=0.764, *I*^2^=0.0% | *P*=0.152 | High | *P*=0.602 | *P*=0.996 |
| PSK plus UFT vs UFT alone | 3(408/203) | 2.27(1.51,3.43)* | *P*=0.560, *I*^2^=0.0% |  | High | *P*=0.014^‡^ | *P*=0.032^‡^ |
|  |  |  |  |  |  |  |  |
| ***3-year DFS*** |  |  |  |  |  |  |  |
| Total | 11(1216/1035) | 1.58(1.30,1.92)* | *P*=0.515, *I*^2^=0.0% |  | High | *P*=0.102 | *P*=0.002^‡^ |
| Cancer type |  |  |  |  |  |  |  |
| CRC | 8(948/750) | 1.59(1.23,2.05)* | *P*=0.319, *I*^2^=14.2% | *P*=0.950 | High | *P*=0.621 | *P*=0.365 |
| GC | 3(268/285) | 1.59(1.10,2.32)* | *P*=0.601, *I*^2^=0.0% |  | High | *P*=0.117 | *P*=0.046^‡^ |
| Treatment arms |  |  |  |  |  |  |  |
| PSK plus 5-Fu vs 5-Fu alone | 5(665/695) | 1.51(1.16,1.97)* | *P*=0.764, *I*^2^=0.0% | *P*=0.526 | High | *P*=0.602 | *P*=0.306 |
| PSK plus UFT vs UFT alone | 3(408/203) | 2.27(1.51,3.43)* | *P*=0.560, *I*^2^=0.0% |  | High | *P*=0.050 | *P*=0.061 |
|  |  |  |  |  |  |  |  |
| ***4-year DFS*** |  |  |  |  |  |  |  |
| Total | 11(1216/1035) | 1.73(1.43,2.10)* | *P*=0.461, *I*^2^=0.0% |  | High | *P*=0.014^‡^ | *P*=0.000 |
| Cancer type |  |  |  |  |  |  |  |
| CRC | 8(948/750) | 1.87(1.46,2.40)* | *P*=0.296, *I*^2^=17.0% | *P*=0.397 | High | *P*=0.026^‡^ | *P*=0.044 |
| GC | 3(268/285) | 1.50(1.04,2.15)* | *P*=0.790, *I*^2^=0.0% |  | High | *P*=0.117 | *P*=0.180 |
| Treatment arms |  |  |  |  |  |  |  |
| PSK plus 5-Fu vs 5-Fu alone | 5(665/695) | 1.41(1.11,1.80)* | *P*=0.733, *I*^2^=0.0% | *P*=0.051 | High | *P*=0.602 | *P*=0.663 |
| PSK plus UFT vs UFT alone | 3(408/203) | 2.44(1.68,3.52)* | *P*=0.869, *I*^2^=0.0% |  | High | *P*=0.014^‡^ | *P*=0.011^‡^ |
|  |  |  |  |  |  |  |  |
| ***5-year DFS*** |  |  |  |  |  |  |  |
| Total | 10(1116/917) | 1.74(1.43,2.11)* | *P*=0.683, *I*^2^=0.0% |  | High | *P*=0.037^‡^ | *P*=0.000 |
| Cancer type |  |  |  |  |  |  |  |
| CRC | 8(948/750) | 1.78(1.43,2.21)* | *P*=0.574, *I*^2^=0.0% |  | High | *P*=0.048^‡^ | *P*=0.005^‡^ |
| GC | 2(168/167) | 1.57(1.00,2.46)* | *P*=0.435, *I*^2^=0.0% |  | Moderate | *P*=0.317 | *-* |
| Treatment arms |  |  |  |  |  |  |  |
| PSK plus 5-Fu vs 5-Fu alone | 4(565/577) | 1.48(1.14,1.91)* | *P*=0.500, *I*^2^=0.0% |  | High | *P*=0.602 | *P*=0.788 |
| PSK plus UFT vs UFT alone | 3(408/203) | 2.03(1.42,2.91)* | *P*=0.896, *I*^2^=0.0% |  | High | *P*=0.174 | *P*=0.029^‡^ |
|  |  |  |  |  |  |  |  |
| ***6-year DFS*** |  |  |  |  |  |  |  |
| Total | 5(487/487) | 1.49(1.11,2.00)* | *P*=0.374, *I*^2^=5.8% |  | High | *P*=0.355 | *P*=0.000 |
| Cancer type |  |  |  |  |  |  |  |
| CRC | 3(319/320) | 1.81(0.95,3.45) | *P*=0.122, *I*^2^=52.5%^#^ | *P*=0.746 | Moderate | *P*=0.602 | *P*=0.083 |
| GC | 2(168/167) | 1.45(0.93,2.27) | *P*=0.851, *I*^2^=0.0% |  | High | *P*=0.317 | *-* |
|  |  |  |  |  |  |  |  |
| ***7-year DFS*** |  |  |  |  |  |  |  |
| Total | 5(487/487) | 1.66(1.11,2.48)* | *P*=0.154, *I*^2^=40.1% |  | High | *P*=0.064 | *P*=0.000 |
| Cancer type |  |  |  |  |  |  |  |
| CRC | 3(319/320) | 2.15(0.92,5.04) | *P*=0.037, *I*^2^=69.8%^#^ | *P*=0.599 | Moderate | *P*=0.117 | *P*=0.093 |
| GC | 2(168/167) | 1.45(0.93,2.27) | *P*=0.851, *I*^2^=0.0% |  | High | *P*=0.317 | *-* |

C, control group; CRC,colorectal cancer CT,chemotherapy; DFS,disease free survival; EPC,Esophageal Cancer; GC,gastric cancer; IT, immunochemotherapy; OS,overall survival; P, PSK group; PSK, Polysaccharide K; RT，radiotherapy; UFT, Tegafur/uracil.

^*^Result with significant differences; ^#^Substantial heterogeneity; ^†^Factors could be an important source of heterogeneity; ^‡^Publication bias.
